# Effect of GDMT Optimization on KDIGO Risk Categories in HFrEF: Analysis of the VIENNA-HF Registry

**DOI:** 10.1016/j.jacadv.2026.102822

**Published:** 2026-06-17

**Authors:** Noel G. Panagiotides, Annika Weidenhammer, Suriya Prausmüller, Henrike Arfsten, Daniel Engler, Elias Stix, Georg Spinka, Gregor Heitzinger, Fanny Hoffmann, Cornelia Gabler, Guido Strunk, Philipp E. Bartko, Georg Goliasch, Christian Hengstenberg, Martin Hülsmann, Noemi Pavo

**Affiliations:** a Department of Internal Medicine II, Clinical Division of Cardiology, Medical University of Vienna, Vienna, Austria; b Complexity Research, Vienna, Austria

**Keywords:** advanced heart failure, albuminuria, HFrEF, KDIGO, kidney failure

## Abstract

**Background:**

Concerns about worsening renal function often limit the use of guideline-directed medical therapy (GDMT) in heart failure (HF) with reduced ejection fraction (HFrEF). Although changes in estimated glomerular filtration rate (eGFR) are commonly monitored during GDMT up-titration, the added value of urine albumin-to-creatinine ratio (UACR) and Kidney Disease: Improving Global Outcomes (KDIGO) risk categories remains less studied.

**Objectives:**

The objectives of the study were to assess changes in eGFR, UACR, and KDIGO risk categories following GDMT optimization in HFrEF and to evaluate their association with renal and HF outcomes.

**Methods:**

Consecutive HFrEF (n = 234) outpatients with baseline and 12-month follow-up data were analyzed from the VIENNA-HF registry. KDIGO risk trajectories and their association with renal and HF outcomes were assessed.

**Results:**

At follow-up, GDMT dosages significantly increased. eGFR declined (60 vs 56 mL/min/1.73 m^2^; *P* = 0.007), whereas UACR improved (31 vs 24 mg/g; *P* = 0.003). 56%, 24%, and 20% of patients exhibited stable, worsening, or improved KDIGO trajectories. UACR contributed to over half of all reclassifications and was the sole determinant in 35% of cases. Event rates increased stepwise across baseline KDIGO risk classes (renal events: 4% vs 12% vs 15% vs 27%; *P* < 0.001; HF events: 19% vs 29% vs 37% vs 56%; *P* < 0.001, 2-years estimate). Similarly and worsening KDIGO trajectories were associated with higher event rates (renal events: 6% vs 15% vs 21%; *P* = 0.034; HF events: 11% vs 43% vs 46%; *P* = 0.007, 2-year estimate).

**Conclusions:**

KDIGO risk trajectories vary substantially during GDMT optimization in HFrEF. UACR provides complementary information beyond eGFR and may improve risk stratification for renal and HF outcomes.

Heart failure (HF) with reduced ejection fraction (HFrEF) and chronic kidney disease (CKD) are closely interrelated conditions that frequently coexist and mutually exacerbate disease progression.^1^ Even in early CKD stages, patients face a significantly increased risk of cardiovascular events.^2^

To assess kidney function, the Kidney Disease: Improving Global Outcomes (KDIGO) guidelines recommend using two complementary biomarkers: estimated glomerular filtration rate (eGFR), indicating filtration capacity, and urine albumin-to-creatinine ratio (UACR), reflecting glomerular permeability.^3^ Both markers independently predict CKD progression and cardiovascular outcomes, and together they form the basis for KDIGO risk classification, guiding prognosis and management.^2^, ^3^, ^4^

In patients with HFrEF, nearly 50% meet the diagnostic criteria for significant CKD, defined as eGFR <60 mL/min/1.73 m^2^, which is associated with worse clinical outcomes, including higher rates of HF hospitalizations and cardiovascular mortality.^5^^,^^6^ Despite the proven benefits of guideline-directed medical therapy (GDMT) on both cardiovascular and renal endpoints, its use remains limited in patients with advanced CKD, due to safety concerns and sparse evidence based on the underrepresentation in clinical trials.^5^, ^6^, ^7^, ^8^, ^9^

A particular barrier to therapy initiation or intensification is the early decline in eGFR often observed with specific GDMT agents, particularly renin-angiotensin system inhibitors (RASis), mineralocorticoid receptor antagonists (MRAs), and sodium-glucose cotransporter 2 inhibitors (SGLT2is). However, this early eGFR drop typically represents hemodynamic rather than structural renal changes, and may in fact be associated with improved long-term cardiorenal outcomes.^9^^,^^10^ In contrast, UACR tends to decrease under these therapies, potentially indicating nephroprotective effects, whereas angiotensin receptor-neprilysin inhibitor (ARNI) use may slightly increase UACR.^10^^,^^11^

These divergent trajectories of eGFR (often numerical worsening) and UACR (mostly improving) highlight their differing physiological responses to GDMT and suggest that a dual assessment may offer deeper insights into renal adaptation during HFrEF treatment. Although eGFR trends are commonly reported in HF trials, longitudinal data on UACR remain limited. Moreover, the prognostic significance of simultaneous changes in eGFR and UACR, combined as KDIGO risk class, during GDMT optimization in HFrEF remains unclear.

This study aimed to evaluate changes in eGFR, UACR, and KDIGO risk class over 12 months in patients with HFrEF undergoing comprehensive GDMT up-titration. Specifically, shifts in KDIGO risk classification and the association of the trajectories with renal and HF outcomes will be assessed.

## Methods

### Patient population

The VIENNA-HF registry has prospectively enrolled consecutive outpatients with HFrEF at the Vienna General Hospital since 2015. Eligibility criteria for referral to the outpatient clinic include a documented left ventricular ejection fraction ≤35% by echocardiography and a significantly elevated N-terminal-pro-brain natriuretic peptide (NT-proBNP) level >1,500 pg/mL.^7^ The clinic specializes in therapy GDMT optimization, device therapy, and advanced HF management (eg, heart transplantation or long-term mechanical circulatory support such as left ventricular assist device).

Clinical data, patient history, cardiac imaging, medication (including dosages), and laboratory values are routinely documented at baseline and all follow-up visits. All-cause and unplanned HF-related hospitalizations are systematically recorded during follow-up. Mortality data are obtained from the Austrian Death Registry.

For this study, we included patients enrolled between January 2021 and December 2024 who had both baseline and 12-month follow-up visits (defined as 12 ± 6 months; 12M visit) with complete data on eGFR and UACR analysis. Routine follow-up visits were scheduled pragmatically reflecting real-world care, with more frequent visits during the first 3 months after first presentation for GDMT up-titration and more widely spaced visits thereafter. For the planned analysis, a follow-up (FUP) visit was defined as a visit between 6 to 18 months after first presentation, whereas in case that more visits were present, the closest to 12 months was selected. The time frame ensures completed up-titration and sufficient time for treatment effects. The study was conducted in accordance with the Declaration of Helsinki and approved by the local ethics committee (EK 1612/2015 & 1579/2025). All participants provided written informed consent.

### Assessing GDMT in HF

The precise use and dosing of HF medications—beta-blockers (BBs), RASi, MRA, and SGLT2i—were recorded at each visit. To allow for standardized comparisons, drug dosages were expressed as a percentage of the respective target doses (TDs). Nonuse of a specific drug was documented as 0%. The overall intensity of quadruple therapy was calculated as the mean percentage across all 4 drug classes as^7^^,^^8^^,^^12^

$$quadruple-therapy\;TD\%=\frac{1}{4}\cdot \left(\text{BB}\;\text{TD}\%+\text{RASi}\;\text{TD}\%+\text{MRA}\;\text{TD}\%+\text{SGLT}2i\;\text{TD}\%\right)$$

### KDIGO risk categories

eGFR and UACR values were assessed at baseline and at the 12-month visit and grouped according to the updated 2024 KDIGO risk guidelines, defined as.

• eGFR: G1 (≥90 mL/min/1.73 m^2^), G2 (60-89), G3a (45-59), G3b (30-44), G4 (15-29), G5 (<15)
• UACR: A1 (<30 mg/g), A2 (30-300 mg/g), A3 (>300 mg/g).^3^

KDIGO CKD risk categories were then defined using combinations of the respective eGFR and UACR groups.

• Low risk: G1A1, G2A1
• Moderately increased risk: G1A2, G2A2, G3aA1
• High risk: G1A3, G2A3, G3aA2, G3bA1
• Very high risk: G3aA3, G3bA2/A3, G4A1-3, G5A1-3.^3^

### Renal outcomes and HF outcomes

Renal events were defined as hospitalizations due to acute kidney injury (AKI), progression to kidney failure, or the initiation of renal replacement therapy (RRT). According to the KDIGO definition, AKI was defined as a reversible and clinically relevant rise in serum creatinine (≥0.5 mg/dL or 20% creatinine increase) and/or oliguria. Acute kidney failure was defined as hospitalization for eGFR <15 mL/min/1.73 m^2^ without recovery. RRT was defined as initiation of long-term RRT, that is, sustained use of dialysis or kidney transplantation. HF outcomes included all-cause mortality and HF hospitalization.

### Statistical analysis

Baseline characteristics were summarized using medians with IQRs for continuous variables and counts with percentages for categorical variables.

To evaluate GDMT up-titration over time, drug dosages (per class and overall) were compared using the paired Wilcoxon signed-rank test. For visualization, dosages were grouped into clinically relevant categories, that is, zero (0%), low (>0%-<50%), medium (≥50%-<90%), and high (≥90%) dosages of TD%.^12^ Changes in HF drug dosage groups were visualized by histograms. Histograms and the Kolmogorov-Smirnov test were used to test data distribution.

The change in biomarker levels between baseline and 12 months was compared for eGFR and UACR, but also NT-proBNP and renin. Changes in eGFR and UACR were also visualized and compared for the respective groups separately, that is, G1-G5 and A1-A3. For all comparisons, the paired Wilcoxon signed-rank test was used. NYHA functional class and NT-proBNP levels at baseline and follow-up were compared using the marginal homogeneity test and the paired Wilcoxon signed-rank test for the total cohort.

KDIGO risk classes were assessed according to eGFR/UACR groups at baseline and at the 12-month visit. The proportion of patients within the risk classes was indicated in tables. eGFR/UACR-based KDIGO risk transitions from baseline to 12M were illustrated using a color-coded matrix and a Sankey diagram, where stream widths were proportional to the number of patients shifting between risk categories. Changes in KDIGO risk distribution were assessed using the McNemar-Bowker test. In addition, patients were grouped into worsening, improving, or stable KDIGO trajectory groups. In case of KDIGO class change, the responsible parameters, that is, eGFR, UACR, or both, were assessed. Baseline characteristics were compared between the KDIGO trajectory groups. Comparisons of continuous variables were performed using the Kruskal-Wallis test, whereas categorical variables were analyzed with Pearson’s chi-square test. Furthermore, NYHA functional class and NT-proBNP levels were compared between baseline and FUP stratified according to KDIGO risk trajectories. Both baseline KDIGO class as well as KDIGO trajectories were evaluated for associations with renal and HF outcomes. For baseline KDIGO risk categories, time-to-event was calculated from the baseline visit, whereas for analyses of KDIGO trajectories, follow-up started at the 12-month visit. Cox proportional hazards regression analyses (univariable and multivariable) were performed to assess the association of baseline KDIGO risk categories and KDIGO risk trajectories with renal and HF outcomes. Baseline KDIGO categories, that is low, moderate, high and very-high, and KDIGO trajectories, that is, stable, worsened, and improved, were modeled as categorical variables, with predefined reference groups (low risk for baseline KDIGO categories and stable for KDIGO trajectories). In a clinical risk-model we adjusted for key baseline clinical covariates as age, sex, BMI, NYHA functional class, and systolic blood pressure. In addition, a data-driven model was generated using a stepwise approach with forward and backward selection. We tested and satisfied the proportional hazards assumption in all cases using Schoenfeld residuals. Kaplan-Meier plots were used to illustrate event-free survival, and groups were compared using the log-rank test for trend.

SPSS (IBM, version 25.0), R (R Core Team 2025, version 4.5.2) and GraphPad Prism (GraphPad Software, version 10) were used to perform statistical analyses and create the figures. A 2-tailed *P* value <0.05 was deemed statistically significant.

## Results

### Study population

In total, patients completed a median of 6 visits (5-7) within 18 months after first presentation. The median follow-up from baseline to the selected 12M FUP visit was 11.3 months (9.5-12.6) Baseline characteristics of the study cohort (n = 234) are summarized in Table 1. The median age was 64 years (51-73), and 27% of patients were females. Patients were distributed across NYHA functional classes as follows: I (14%), II (51%), and III/III+ (35%). The median NT-proBNP at baseline was 1930 pg/mL (684-4954). At baseline, 90%, 88%, 73%, and 67% of patients received BB, RASi, MRA, and SGLT2i therapy. 65%, 52%, and 70% of patients were receiving at least 50% of the target dosages for BB, RASi, and MRA, respectively.

**Table 1:** Baseline Characteristics

	Total Cohort (N = 234)	KDIGO-Trajectory Stable (n = 130)	KDIGO-Trajectory Improved (n = 48)	KDIGO-Trajectory Worse (n = 56)	*P* Value
Basic demographics					
Age, y, median (IQR)	64 (51-73)	62 (49-73)	64 (47-74)	64 (57-72)	0.722
Female, n (%)	62 (27%)	31 (23.8%)	15 (31.3%)	16 (28.6%)	0.563
BMI, kg/m^2^, median (IQR)	26.4 (23.3-30.1)	26.7 (23.6-30.0)	25.4 (22.4-29.5)	26.6 (23.2-31.1)	0.365
Systolic BP, mm Hg, median (IQR)	120 (106-140)	120 (110-140)	120 (109-135)	120 (100-134)	0.452
Diastolic BP, mm Hg, median (IQR)	76 (70-85)	77 (70-86)	79 (68-85)	75 (66-85)	0.728
Heart rate, beats/min (IQR)	71 (62-82)	70 (60-79)	70 (62-87)	73 (62-82)	0.348
NYHA functional class, n (%)					0.362
I	33 (14%)	14 (11.1%)	12 (25.5%)	7 (12.7%)	
II	116 (51%)	67 (53.2%)	21 (44.7%)	28 (50.9%)	
III/IV	79 (35%)	45 (35.7%)	14 (29.8%)	20 (36.3%)	
Comorbidities					
Arterial hypertension, n (%)	96 (41%)	48 (36.9%)	23 (47.9%)	23 (41.1%)	0.189
CAD, n (%)	123 (53%)	67 (51.5%)	21 (43.8%)	35 (62.5%)	0.152
Atrial fibrillation, n (%)	89 (38%)	47 (36.2%)	21 (43.8%)	21 (37.5%)	0.648
Diabetes mellitus type II, n (%)	73 (31%)	39 (30.0%)	13 (27.1%)	21 (37.5%)	0.472
Chronic kidney disease, n (%)	116 (50%)	63 (48.5%)	33 (68.6%)	20 (35.7%)	0.003
COPD, n (%)	21 (9%)	11 (8.5%)	5 (10.4%)	5 (8.9%)	0.921
PAD, n (%)	27 (12%)	12 (9.2%)	2 (4.2%)	13 (23.2%)	0.005
Carotid artery disease, n (%)	19 (8%)	11 (8.5%)	2 (4.2%)	6 (10.7%)	0.465
Stroke, n (%)	18 (8%)	11 (8.5%)	3 (6.3%)	4 (7.1%)	0.873
Any malignant disease, n (%)	39 (17%)	24 (18.5%)	10 (20.8%)	5 (8.9%)	0.191
Medication					
Beta-blocker, n (%)	211 (90%)	115 (88.5%)	44 (91.7%)	52 (92.9%)	0.605
Recommended target dose ≥50%, n (%)	151 (65%)	84 (64.6%)	35 (72.9%)	32 (57.1%)	0.245
RASi, n (%)	205 (88%)	113 (86.9%)	42 (87.5%)	50 (89.3%)	0.904
Recommended target dose ≥50%, n (%)	122 (52%)	67 (51.5%)	27 (56.3%)	28 (50.0%)	0.800
MRA, n (%)	171 (73%)	88 (67.7%)	35 (72.9%)	48 (85.7%)	0.040
Recommended target dose ≥50%, n (%)	164 (70%)	85 (65.4%)	34 (70.8%)	45 (80.4%)	0.122
SGLT2i, n (%)	156 (67%)	79 (60.8%)	33 (68.8%)	44 (78.6%)	0.058
Diuretics, n (%)	117 (50%)	65 (50%)	24 (50%)	28 (50%)	0.967
Devices					
ICD, n (%)	79 (34%)	43 (33.1%)	19 (39.6%)	17 (30.4%)	0.593
CRT, n (%)	46 (20%)	28 (21.5%)	6 (12.5%)	12 (21.4%)	0.375
Laboratory parameters					
NT-proBNP pg/mL, median (IQR)	1930 (684-4,954)	1797 (603-6,088)	1965 (779-3,850)	2,163 (1,102-3,719)	0.924
Creatinine, mg/dL, median (IQR)	1.18 (0.97-1.64)	1.17 (0.95-1.94)	1.31 (1.09-1.63)	1.14 (0.95-1.28)	0.102
eGFR, mL/min/1.73 m^2^, median (IQR)	60.4 (39.8-74.9)	63.4 (32.6-79.2)	53.4 (39.2-65.8)	62.6 (52.3-71.8)	0.212
UACR, mg/g, median (IQR)	30.5 (11.6-101.6)	30.2 (11.1-141.9)	64.4 (35.5-176.3)	21.9 (9.8-34.2)	**<0.001**
Potassium, mmol/L, median (IQR)	4.7 (4.4-4.9)	4.7 (3.4-5.0)	4.62 (4.4-4.9)	4.68 (4.4-5.1)	0.613
BUN, mg/dL, median (IQR)	21.2 (15.9-31.9)	20.2 (15.1-35.9)	22.9 (17.6-32.4)	22.4 (16.0-26.1)	0.591
Sodium, mmol/L, median (IQR)	140 (138-142)	140 (138-141)	140 (138-142)	140 (138-142)	0.614
BChE, kU/L, median (IQR)	7.3 (5.9-8.4)	7.4 (5.8-8.7)	7.4 (6.1-8.4)	6.8 (6.0-7.7)	0.211
AST (GOT), U/L, median (IQR)	25 (20-31)	25 (19-30)	24 (21-32)	25 (21-32)	0.646
ALT (GPT), U/L, median (IQR)	26 (18.3-37.8)	26 (18-39)	26 (20-35)	26 (19-33)	0.985
GGT, U/L, median (IQR)	38 (25-72)	37 (23-75)	38 (25-63)	42 (32-70)	0.194
Total bilirubin, mg/dL, median (IQR)	0.53 (0.38-0.76)	0.52 (0.39-0.77)	0.54 (0.36-0.80)	0.54 (0.37-0.73)	0.777
Ferritin, ng/mL, median (IQR)	1,601 (89-281)	173 (101-285)	116 (51-205)	194 (112-312)	0.055
TSAT, %, median (IQR)	22 (14.3-30.5)	23.6 (14.1-31.4)	19.9 (13.6-31.2)	22.0 (17.3-28.4)	0.670
Hemoglobin, g/dL, median (IQR)	13.7 (11.8-15)	13.4 (11.6-15.2)	13.6 (11.6-14.9)	14.0 (12.9-14.9)	0.524
Leukocyte count, G/L, median (IQR)	7.8 (6.5-9.6)	8.06 (6.69-9.73)	7.26 (6.14-9.33)	7.85 (6.58-9.37)	0.284
Triglycerides, mg/dL, median (IQR)	114 (82-161)	115 (82-159)	112 (75-169)	123 (92-165)	0.467
Total cholesterol, mg/dL, median (IQR)	139 (112-178)	131 (110-175)	150 (114-180)	142 (115-181)	0.378
HbA1c, %, median (IQR)	6.0 (5.5-6.5)	6.0 (5.5-6.5)	5.7 (5.3-6.2)	6.1 (5.7-6.7)	**0.027**
CRP, mg/dL, median (IQR)	0.25 (0.08-0.65)	0.23 (0.07-0.64)	0.18 (0.08-0.56)	0.32 (0.11-0.83)	0.155

Bold values indicate statistical significance (*P* < 0.05).

ALT = alanine aminotransferase; AST = aspartate aminotransferase; BChE = butyrylcholinesterase; BMI = body mass index; BP = blood pressure; BUN = blood urea nitrogen; CAD = coronary artery disease; COPD = chronic obstructive pulmonary disease; CRP = C-reactive protein; CRT = cardiac resynchronization therapy; eGFR = estimated glomerular filtration rate; GOT = Glutamate Oxaloacetate Transaminase; GGT = gamma-glutamyl transferase; GPT = Glutamate Pyruvate Transaminase; HbA1c = hemoglobin A1c; ICD = implantable cardioverter defibrillator; KDIGO = Kidney Disease: Improving Global Outcomes; MRA = mineralocorticoid receptor antagonist; NT-proBNP = N-terminal-pro-brain natriuretic peptide; PAD = peripheral artery disease; RASi = renin angiotensin system inhibitor; SGLT2i = sodium-glucose cotransporter 2 inhibitor; TSAT = transferrin saturation; UACR = urine albumin-to-creatinine ratio.

### GDMT optimization of HF medication

**Figure 1:**
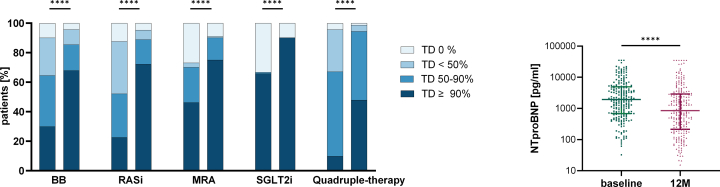
**Up-Titration of Guideline-Directed Medical Therapy and Change in N-Terminal-Pro-Brain Natriuretic Peptide** The percentage of patients achieving the target dose (TD) of beta-blockers (BB), renin-angiotensin system inhibitors (RASi), mineralocorticoid receptor antagonists (MRA), sodium-glucose co-transporter-2 inhibitors (SGLT2i), and quadruple-therapy, as well as the difference in NT-proBNP at baseline (left) and after 12 months (right). Achieved TDs of the respective medications and NTproBNP values were compared using the nonparametric paired Wilcoxon signed-rank test. NT-proBNP - N-terminal-pro-brain natriuretic peptide; ∗*P* < 0.05; ∗∗*P* < 0.01; ∗∗∗*P* < 0.001; ∗∗∗∗*P* < 0.0001.

The achieved TDs for all HF drug classes at 12M compared to baseline (BL) are shown in Figure 1. A significant increase in TDs could be observed for all drug classes at 12M (*P* < 0.001 for all comparisons for the continuous variable of TDs). The majority of patients received ≥50% and ≥90% of the recommended TDs at 12M (for ≥50%: 85%, 89%, 87%, 90%, and 95%; and for ≥90%: 68%, 72%, 75%, 90%, and 48% for BB, RASi, MRA, SGLT2i, and quadruple-therapy, respectively).

GDMT implementation was accompanied by a significant decrease in NT-proBNP (BL vs 12M: 1930 [684-4954] vs 838 [213-2,878] pg/mL; *P* < 0.001) and an increase in active renin concentration (BL vs 12M: 146 [29-679] vs 202 [36-742] μlU/mL; *P* = 0.015).

### Change in eGFR, UACR, and KDIGO risk trajectories

**Figure 2:**
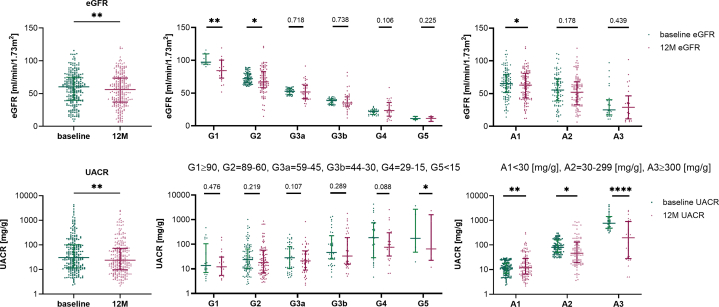
**Trajectories of Estimated Glomerular Filtration Rate and Urine Albumin-to-Creatinine Ratio Across Chronic Kidney Disease Categories** The median eGFR and UACR values with 95% CIs for both time points and across eGFR and UACR categories are depicted. Changes from baseline to 12M are shown across the CKD categories according to eGFR and UACR: eGFR (mL/min/1.73 m^2^): G1 ≥90, G2 60 to 89, G3a 45 to 59, G3b 30 to 44, G4 15 to 29, and G5 <15; and UACR (mg/g): A1 <30, A2 30 to 300, and A3 >300. eGFR and UACR values were compared using the paired Wilcoxon signed-rank tests. ∗*P* < 0.05; ∗∗*P* < 0.01; ∗∗∗∗*P* < 0.0001. eGFR = estimated glomerular filtration rate; UACR = urine albumin-to-creatinine ratio.

Figure 2 shows changes in eGFR and UACR across the entire cohort and within KDIGO subgroups. Overall, eGFR significantly declined, whereas UACR improved following GDMT optimization (eGFR: 60 [40-75] vs 56 [37-74] mL/min/1.73 m^2^; *P* = 0.007; UACR: 31 [12-102] vs 24 [10-73] mg/g; *P* = 0.003). The reduction in eGFR was most pronounced in patients with initially good renal function (*P* = 0.004 for G1 and *P* = 0.011 for G2 and *P* = 0.027 for A1). In contrast, improvement in UACR was observed for patients with worse renal function (*P* = 0.043 for G5, *P* = 0.024 for A2, and *P* < 0.001 for A3).

**Figure 3:**
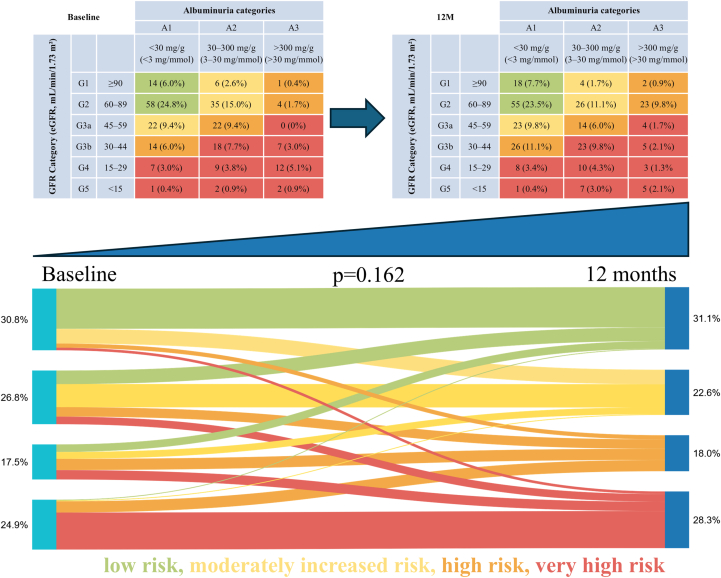
**Distribution of Kidney Disease: Improving Global Outcomes Risk Categories After Guideline-Directed Medical Therapy Optimization** The distribution and change of patient risk categories, as defined by the KDIGO CKD 2024 classification, at baseline and after 12 months are shown. Classification via eGFR and UACR. Green: low risk, yellow: moderately increased risk, orange: high risk, red: very high risk. The McNemar-Bowker Test assessed the overall change in KDIGO categories. The *P* value is indicated in the respective figure. Abbreviation as in Figure 2.

**Figure 4:**
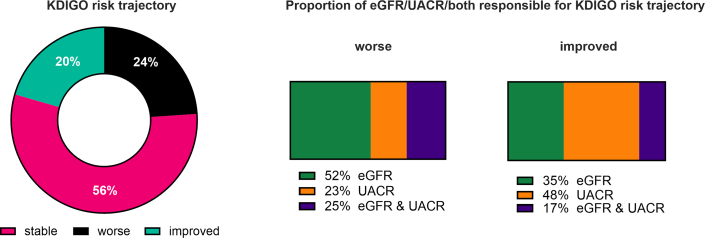
**Kidney Disease: Improving Global Outcomes Risk Trajectory and the Contribution of Renal Biomarkers** (Left) Distribution of KDIGO risk class trajectories after 12 months. (Right) Contributions of renal biomarkers - eGFR, UACR, or both - to changes in KDIGO risk class. KDIGO = Kidney Disease: Improving Global Outcomes; other abbreviations as in Figure 2.

Although 104 (44%) of patients showed a transition across KDIGO 2024 risk categories on GDMT up-titration, the distribution across categories in total remained unchanged (*P* = ns) (Figure 3). The distribution of KDIGO risk trajectories and whether eGFR, UACR, or both led to a transition in the KDIGO risk class is shown in Figure 4. eGFR, UACR, and both biomarkers were responsible for 52%, 23%, and 25% in case of worsening, and for 35%, 48%, and 17% in case of improving KDIGO risk. UACR levels were involved in 58/104 (56%) cases of KDIGO risk change, and UACR was the sole biomarker indicating a change in 36/104 (35%) cases. Table 1 shows the comparison of baseline characteristics of patients with different KDIGO risk trajectories. Almost all parameters, including age and gender, but most importantly, NT-proBNP and baseline HF medication, were comparable between groups (*P* = ns for all). eGFR was similarly comparable (*P* = ns), whereas UACR was somewhat higher in patients showing KDIGO risk class improvement (*P* < 0.001).

Supplemental Figure 1 shows the changes in NT-proBNP and NYHA functional class for the total cohort and according to KDIGO risk trajectories. Over 12 months during GDMT optimization, NT-proBNP decreased significantly in the overall cohort (*P* < 0.001) and also across all KDIGO trajectories (stable and improved: *P* < 0.001; worsened: *P* = 0.007). NYHA functional class improved overall (*P* < 0.001) and in the stable KDIGO group (*P* < 0.001), with nonsignificant trends toward improvement in the improved (*P* = 0.063) and worsened (*P* = 0.083) KDIGO groups.

### Adverse renal events and HF prognosis

The median FUP time was 22 months (16-27) from baseline and 11 months (5-15) after the 12M visit. During FUP, a total of 29 (12%) patients from baseline and 16 (7%) patients after GDMT optimization experienced adverse renal events (from baseline: AKI n = 23, acute kidney failure n = 3, initiation of new RRT n = 3; from the 12M visit: AKI n = 13, acute kidney failure n = 1, initiation of new RRT n = 2). A total of 79 (34%) patients from baseline and 52 (22%) patients after GDMT optimization experienced hospitalization or death.

The median serum creatinine increase in the AKI patients was +1.6 (1.3-3.4) mg/dL or +94% (70-144), reaching ≥1.9× baseline creatinine. Hyperkalemia (potassium >5.0 mmol/L) was present in 47/234 (20%) patients at baseline and in 78/234 (33%) patients at 12M. A simultaneous increase in the use of potassium-binding therapy was observed (3.4% vs 17.1%).

**Figure 5:**
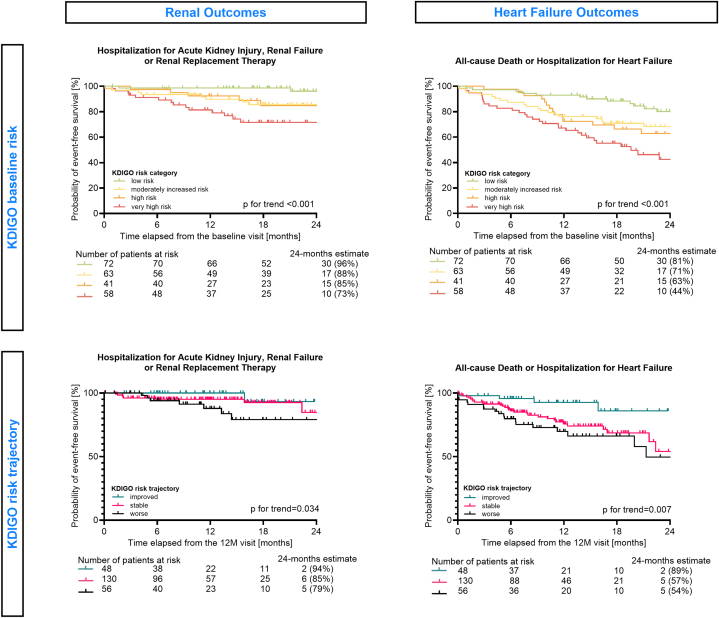
**Renal and Heart Failure Outcomes by Kidney Disease: Improving Global Outcomes Risk and Trajectories** Depicted is the combined renal endpoint (left) and the combined HF endpoint of all-cause death or first heart failure hospitalization (right) for the KDIGO baseline risk (top) and KDIGO risk trajectory (below), the log-rank test for trend compared data. The *P* value is indicated in the respective figure. Abbreviation as in Figure 4.

Figure 5 shows the Kaplan-Meier curve analysis for renal and HF outcomes for the baseline KDIGO risk categories. Baseline KDIGO risk categories were clearly associated with renal events. The event rate increased gradually with increasing KDIGO risk category (2-year estimates for freedom from adverse renal events were 96%, 88%, 85%, and 73%, *P* < 0.001 for trend). Interestingly, similar findings were observed for HF outcomes (2-year estimates for freedom from hospitalization or death 81%, 71%, 63%, and 44%, *P* < 0.001 for trend), respectively. In unadjusted Cox models, when modeled as an ordinal variable and using the low-risk group as reference, HF risk increased stepwise across categories (moderate: HR: 1.93 [0.98-3.91]; *P* = 0.056; high: HR: 2.10 [1.01-4.39]; *P* = 0.048; very high: HR: 3.81 [2.05-7.42]; *P*< 0.001). The association with an increase in risk for these categories remained significant after adjustment for both the clinical model as well as the data driven model.

Kaplan-Meier analyses for KDIGO risk trajectories demonstrated significant differences between groups for both renal (2-year event-free survival: 94%, 85%, and 79%; *P* = 0.034 for trend) and HF outcomes (89%, 57%, and 54%; *P* = 0.007 for trend). In unadjusted Cox models, when modeled as an ordinal variable and using the stable KDIGO trajectory group as reference, a markedly higher renal risk for worsened vs improved trajectories (HR: 6.41 [1.14-119.8]; *P* = 0.033) was observed. For HF outcomes, improvement was associated with lower risk (HR: 0.32 [0.10-0.82]; *P* = 0.015), whereas worsening KDIGO trajectory showed a comparable risk (HR: 1.36 [0.74-2.41]; *P* = 0.314); and additional comparison for worsened vs improved trajectories confirmed a significantly higher risk (HR: 4.18 [1.56-14.48]; *P* = 0.003). The association with an increase in risk for these categories remained significant after adjustment for both the clinical model as well as the data driven model.

## Discussion

This study is the first to examine dynamic changes in eGFR and UACR during comprehensive up-titration of GDMT in patients with HFrEF, and to evaluate the trajectory of KDIGO risk categories in relation to adverse renal events and HF outcomes (Central Illustration). Optimization of all 4 pillars of HF therapy resulted in a modest overall decline in eGFR alongside an improvement in UACR. Patients with near-normal renal function exhibited the most pronounced decrease in eGFR, whereas those with more advanced CKD showed the most significant improvement in UACR. Because of the overall opposing effects of GDMT optimization on renal biomarkers, the distribution of KDIGO risk categories remained essentially unchanged. Both baseline KDIGO risk and subsequent risk trajectories were associated with renal as well as HF outcomes. Notably, UACR contributed to approximately half of the reclassifications, underscoring the importance of incorporating UACR measurement during GDMT up-titration.

**Central Illustration:**
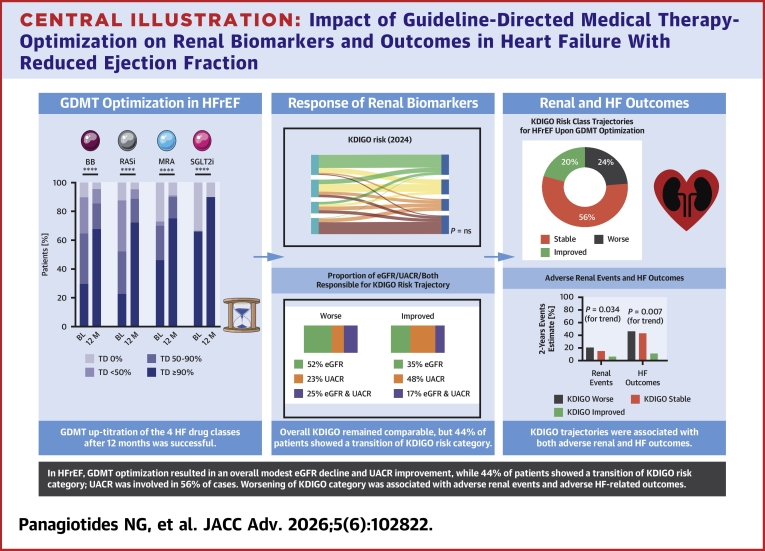
**Impact of Guideline-Directed Medical Therapy-Optimization on Renal Biomarkers and Outcomes in Heart Failure With Reduced Ejection Fraction** Over 12 months, up-titration of beta-blockers, renin-angiotensin system inhibitors, mineralocorticoid receptor antagonists, and sodium-glucose co-transporter-2 inhibitors was successfully achieved. Renal biomarker responses are shown by transitions in KDIGO risk categories from baseline to 12 months and by the relative contribution of changes in eGFR, UACR, or both to KDIGO risk trajectories. Although overall KDIGO risk from baseline to 12 months remained comparable, 44% of patients experienced a change in KDIGO risk category. KDIGO risk trajectories were associated with both adverse renal events and heart failure–related outcomes, with worsening KDIGO risk linked to an increased event burden. BB = beta-blocker; BL = baseline; eGFR = estimated glomerular filtration rate; GDMT = Guideline-Directed Medical Therapy; HF = heart failure; HFrEF = heart failure with reduced ejection fraction; KDIGO = Kidney Disease: Improving Global Outcomes; MRA = mineralocorticoid receptor antagonist; RASi = renin-angiotensin system inhibitor; SGLT2i = sodium-glucose co-transporter-2 inhibitor; TD = target dose; UACR = urine albumin-to-creatinine ratio.

### The cardiorenal axis: eGFR and UACR are complementary biomarkers of renal function

CKD is a prevalent and prognostically significant comorbidity in HFrEF patients. Reduced eGFR—particularly <60 mL/min/1.73 m^2^—is consistently associated with increased mortality, adverse renal, and HF outcomes.^5^^,^^6^ Although some decline in eGFR is expected with aging, patients with HFrEF typically experience more rapid deterioration.^10^^,^^13^^,^^14^ Clinical trial data suggest annual eGFR losses of −1 to −3 mL/min/1.73 m^2^.^10^^,^^14^ Beyond eGFR, albuminuria, or specifically the UACR, is increasingly recognized as a prognostic marker in HFrEF, correlating with disease severity and progression.^11^^,^^15^^,^^16^ A recent analysis from the BIOSTAT-CHF study found that approximately 44% of patients with HFrEF had elevated UACR, which was strongly associated with greater HF severity, increased risk of mortality, and HF hospitalization.^16^

In CKD, the KDIGO guidelines recommend the combined assessment of eGFR and UACR to evaluate renal function and CKD risk.^3^ eGFR reflects the kidney’s filtration capacity and is influenced by hemodynamic and pharmacologic factors, whereas UACR more directly captures structural kidney damage, such as glomerular or tubular injury.^3^^,^^9^ In a large individual-participant data meta-analysis involving over 27,500,000 individuals from 114 global cohorts, both lower eGFR and higher UACR were independently associated with a wide range of adverse outcomes, including mortality, cardiovascular events, kidney failure, and HF.^2^ However, whether the KDIGO classification also applies to patients with HFrEF in predicting renal and HF outcomes has not been intensively investigated yet. KDIGO risk categories were developed for CKD, based on the concept that, in addition to eGFR, UACR more specifically reflects glomerular damage. In the setting of HFrEF, however, both eGFR and UACR can be affected by congestion and tubular stress, as well as by hemodynamic effects intrinsic to the disease itself or mediated by HF therapy, therefore not exclusively reflecting true kidney injury. KDIGO categories in HFrEF may reflect a combination of processes for both diseases and should be interpreted in this context. Nevertheless, KDIGO classification in HFrEF appears to retain important prognostic value. Consistent with this, the data showed that both baseline KDIGO class and changes in KDIGO risk were strongly associated with HF outcomes. The progression to end-stage kidney disease was rare, indicating that KDIGO risk trajectories are more informative for cardiovascular risk stratification than a specific marker of kidney injury.

### Impact of HF therapies on renal biomarkers

The interplay between HF therapies and renal function is complex. Although GDMT confers clear long-term benefits, it often leads to early, hemodynamically driven declines in eGFR.^3^^,^^9^^,^^10^ These changes may reflect therapeutic reductions in intraglomerular pressure rather than structural renal damage.^9^^,^^10^ In contrast, UACR appears more responsive to structural improvement and often declines under effective therapy. Although changes in eGFR are well documented in randomized controlled trials investigating the 4 main drug classes for HFrEF, data on the effect of BB, RASi, MRA, and SGLT2i on UACR are limited and derived mainly from non-HFrEF cohorts. BBs are generally considered kidney-neutral, with negligible impact on eGFR or UACR.^9^ RASi, including ARNI, typically induce modest declines in eGFR but preserve long-term renal function. angiotensin receptor blockers and angiotensin-converting enzyme inhibitors reduce UACR modestly, whereas ARNI use may slightly increase UACR, while still resulting in superior cardiovascular outcomes.^9^^,^^10^^,^^17^^,^^18^ MRAs may slightly lower both eGFR and UACR.^9^^,^^19^ SLGT2is consistently result in early eGFR dips and sustained UACR reductions, while preserving renal function long-term.^9^^,^^20^ Notably, SGLT2i have been shown to slow eGFR decline compared with placebo (empagliflozin/dapagliflozin −0.56/−1.09 vs −2.28/2.86 mL/min/1.73 m^2^).^21^^,^^22^

The data of this study show that the successful implementation of GDMT results not only in lower NT-proBNP but also in higher renin levels. This implicates a reduction of pressure within the afferent arteriole, possibly contributing to kidney protection.^23^ An increase in renin levels upon intensification of HF treatment has been shown in previous studies.^24^^,^^25^ The simultaneous up-titration of all 4 HF drug classes similarly resulted in a modest eGFR decline and UACR reduction, as anticipated from the evidence for each drug separately. Based on the opposing effects of GDMT on renal biomarkers, the distribution of KDIGO risk class remained unchanged.

### Renal function as a therapy limiting factor in HFrEF

Despite frequent clinical concern, modest eGFR declines during GDMT should not prompt premature therapy withdrawal. Previous data of this cohort show that up-titration is feasible across a broad eGFR range, including patients with advanced CKD.^7^ These data show that the most substantial eGFR decline occurred in patients with higher baseline renal function, whereas UACR improved most in those with worse baseline renal function. The median eGFR values in patients with G3a, G3b, and G4 CKD remained stable after 12 months of therapy. With 76%, the majority of patients have a stable or improved KDIGO risk. Only 24% of patients experienced worsening of KDIGO risk class, which was not only associated with adverse renal but also with adverse HF outcomes, suggesting that HF progression may be the primary driver of worsening renal function. Patients with improved KDIGO class had the best outcomes regarding renal or HF endpoints. Regarding baseline characteristics, there was no clear indication of which patients were at risk of worsening KDIGO class during GDMT up-titration. Most importantly, patients with improved, stable, and worsening KDIGO trajectories had comparable levels of HF medication, NT-proBNP, and eGFR at baseline.

### KDIGO risk classification in HFrEF: renal and HF outcomes

Data on the applicability of the KDIGO classification for predicting renal and HF outcomes in HFrEF are limited. A large 2025 U.S. veterans cohort (n = 139,748 HFrEF) showed that KDIGO staging predicted higher 5-year mortality and HF hospitalizations, supporting its role in risk stratification regarding HF progression.^26^ In PARADIGM-HF, approximately 26% of HFrEF patients were categorized as high or very high KDIGO risk; these patients also experienced the highest rates of cardiovascular events, yet sacubitril/valsartan demonstrated consistent efficacy and safety across all KDIGO risk stages.^27^

This is the first study to assess longitudinal changes in eGFR, UACR, and KDIGO risk class over 12 months in patients with HFrEF undergoing comprehensive GDMT up-titration. The data show that KDIGO risk trajectories during GDMT intensification are closely associated with both renal and HF outcomes. Patients who improved in the KDIGO category had the most favorable renal and HF outcomes, whereas those with worsening trajectories experienced the highest rates of adverse renal and HF events. Notably, UACR contributed to 56% of KDIGO risk category changes and was the sole indicative biomarker in 35% of cases. These results suggest that monitoring UACR during GDMT up-titration may refine the assessment of feared complications of adverse renal events and improve the estimation of HF disease progression.

Overall, our results demonstrate that the course of renal biomarkers is highly individual and that the risk of worsening renal function cannot be predicted reliably. We observed substantial variability in eGFR and UACR changes among HFrEF patients during GDMT optimization. Patients with worsening KDIGO trajectories showed higher rates of adverse renal events as well as HF progression, suggesting that deterioration in renal function may in part be driven by HF progression itself. These findings support the concept that patients should undergo GDMT up-titration regardless of baseline renal biomarkers, and that worsening renal parameters should not automatically be interpreted as irreversible kidney injury or an indication to withdraw therapy. UACR provides complementary prognostic information to eGFR and may be a valuable parameter to monitor during GDMT up-titration to refine risk assessment. Larger, prospective studies are warranted to further evaluate the clinical utility of UACR monitoring in HFrEF and to determine whether serial assessment of renal biomarkers can inform treatment decisions and guide follow-up intensity in clinical practice, particularly in the setting of eGFR decline.

### Study Limitations

This study has several limitations. First, inclusion was limited to patients who completed both baseline and 12-month follow-up visits, potentially introducing survivor bias by excluding those with early events. Second, the number of renal events was low, which may have limited the statistical power to detect associations with renal outcomes. Third, generalizability is limited by the single-center design. KDIGO risk categories were not adjusted for baseline eGFR, UACR, or GDMT intensity. Regression-to-the-mean may have influenced observed changes in UACR, particularly among patients with higher baseline values. Nevertheless, the low rate of renal events supports the safety of GDMT intensification, even in patients with impaired kidney function. Future studies in larger, multicenter cohorts are warranted to validate and expand these findings.

## Conclusions

This study highlights the intricate interplay between HFrEF, its medical management, disease progression, and renal biomarkers. Intensification of GDMT is associated with a modest overall decline in eGFR, paralleled by improvements in UACR, whereas trajectories of KDIGO risk show substantial variability. Notably, UACR contributed to approximately half of the observed KDIGO risk transitions and was the sole driver in 35% of cases. Importantly, worsening KDIGO risk over time identifies patients at elevated risk not only for adverse renal outcomes but, more critically, for adverse HF outcomes. These findings suggest that progression of HF is closely intertwined with—if not the primary driver of—deteriorating renal function. Routine monitoring of UACR during the course of HFrEF or during GDMT up-titration may therefore allow more refined risk stratification than relying on eGFR alone. Future studies are warranted to further explore the clinical utility of UACR monitoring in this setting.

Perspectives **COMPETENCY IN MEDICAL KNOWLEDGE:** In patients with HFrEF, a modest decline in eGFR during GDMT intensification is common across most HF drug classes and typically reflects hemodynamic changes rather than structural kidney injury. In this real-world cohort, simultaneous initiation or up-titration of all 4 drug classes resulted in a modest eGFR decline accompanied by UACR improvement. 44% of patients shifted to another KDIGO risk category. Improved, stable, and worsening KDIGO risk trajectories predicted progression to end-stage renal disease as well as adverse HF outcomes. Patients with improvement of the KDIGO risk category have the best prognosis. This finding underscores the close cardiorenal interplay, suggesting that HF progression forms the basis of progressive renal impairment.

**TRANSLATIONAL OUTLOOK**: UACR levels were involved in 56% of KDIGO risk category changes and were the sole responsible biomarker in 35% of cases. This finding supports that UACR offers complementary prognostic information alongside eGFR in HFrEF. Monitoring UACR during the course of HFrEF disease, especially during GDMT up-titration, may assist in monitoring treatment response and help identify patients with the lowest and highest renal and HF risk.

## Funding support and author disclosures

This research was supported by the 10.13039/501100004061Oesterreichische Nationalbank (Austrian Central Bank, Anniversary Fund, project number: 18357). The authors have reported that they have no relationships relevant to the contents of this paper to disclose.
