# Supplementary material for: Effect of GDMT Optimization on KDIGO Risk Categories in HFrEF: Analysis of the VIENNA-HF Registry
Source: JACC Adv. 2026 Jun 17;5(6):102822. doi: 10.1016/j.jacadv.2026.102822 (PMC13308232; doi:10.1016/j.jacadv.2026.102822)
Supplement: Supplemental_Material_-_Figures_1_and_2 [file mmc1.pdf]

Supplemental Material

Table of Contents

Supplemental Figure 1. Changes in NT-proBNP and NYHA class according to KDIGO risk trajectories p. 2

Supplemental Figure 2. Study flowchart p. 3

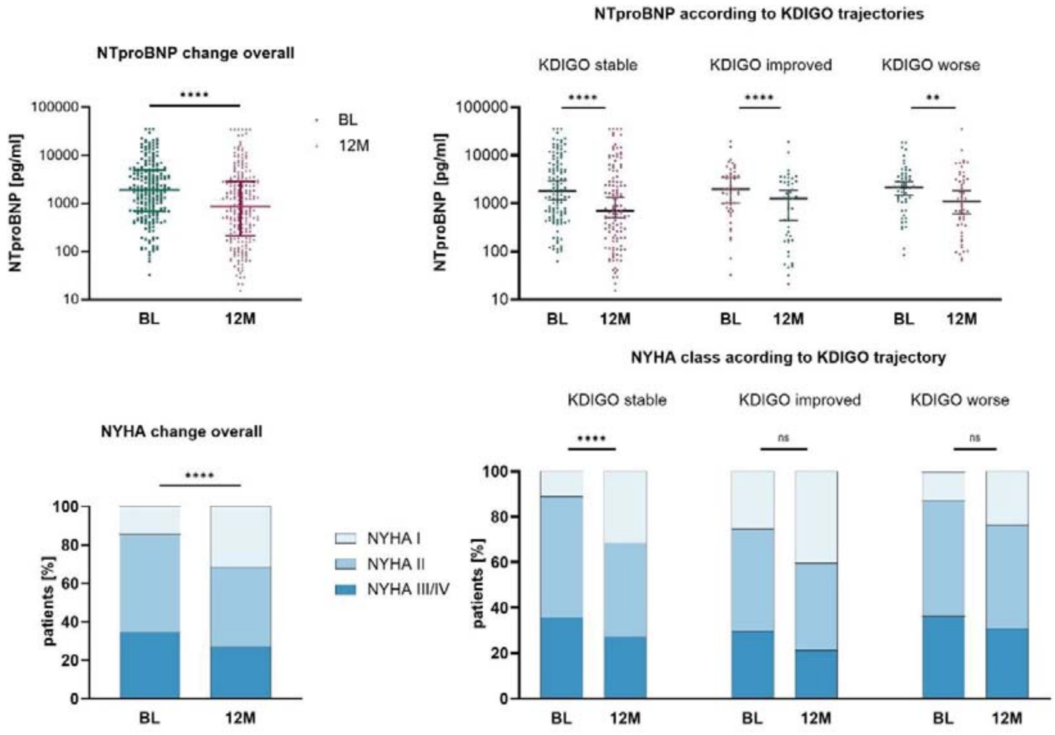

Supplemental Figure 1. Changes in NT-proBNP and NYHA class according to KDIGO risk trajectories. NT-proBNP levels and NYHA functional class at baseline and follow-up are shown for the total cohort and stratified according to KDIGO risk trajectories (stable, improved and worse). NT-proBNP values were compared using the paired Wilcoxon signed-rank test, and NYHA class changes were assessed using the marginal homogeneity test. ns = not significant; * p<0.05; ** p<0.01; *** p<0.001; **** p<0.0001

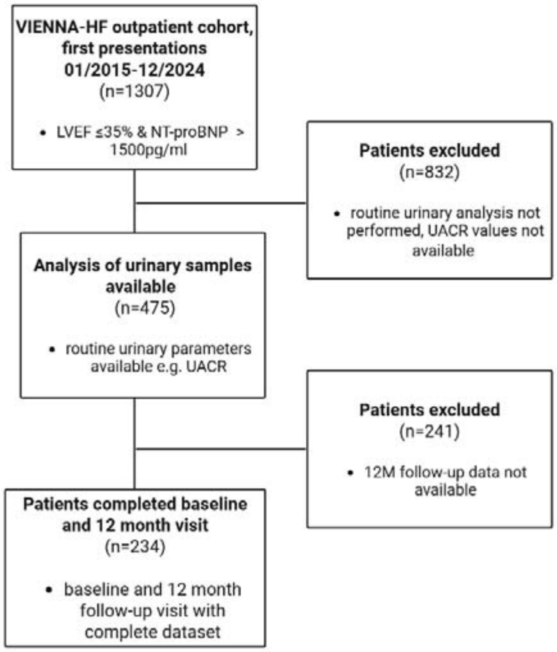

Supplemental Figure 2. Study flowchart. Flow diagram depicting patient selection from the VIENNA-HF registry and the derivation of the final study population.
